# Paratesticular Inflammatory Myofibroblastic Tumor in a Pediatric Patient

**DOI:** 10.1155/2014/303678

**Published:** 2014-05-12

**Authors:** Miriam Harel, John H. Makari

**Affiliations:** ^1^University of Connecticut Health Center, 263 Farmington Avenue, Farmington, CT 06030, USA; ^2^Connecticut Children's Medical Center, 282 Washington Street, Hartford, CT 06106, USA

## Abstract

Although rare, paratesticular inflammatory myofibroblastic tumor (IMT) represents the second most common paratesticular mass after adenomatoid tumor and comprises roughly 6% of such lesions. Only approximately four cases have been reported in patients younger than 18 years of age. We report an incidentally discovered paratesticular IMT in a 17-year-old male successfully treated with wide excision and testis sparing. To our knowledge, no recurrence has been reported after complete excision of paratesticular IMT; however, continued follow-up is recommended.

## 1. Introduction

Inflammatory myofibroblastic tumor (IMT) is an uncommon spindle tumor that may arise in multiple anatomic sites, including the lung, gastrointestinal tract, retroperitoneum, central nervous system, extremities, and the genitourinary tract. Although rare, paratesticular IMT represents the second most common paratesticular mass after adenomatoid tumor and comprises approximately 6% of such lesions [1]. The peak incidence occurs in the third decade of life, and only approximately four cases have been reported in patients younger than 18 years of age [2].

We report an incidentally discovered paratesticular IMT in a 17-year-old male treated with wide excision and testis sparing.

## 2. Case Presentation

A 17-year-old male was referred for urologic evaluation of a right paratesticular mass noted on routine physical examination by his pediatrician. The patient had a history of congenital adrenal hyperplasia, treated with dexamethasone. He had not noticed any scrotal masses and denied any scrotal pain, swelling, or discomfort. The patient denied any history of dysuria, hematuria, urinary tract infection, or sexually transmitted diseases. No history of trauma, fevers, or any other constitutional symptoms was elicited.

Physical examination revealed Tanner stage 5 genitalia with a circumcised phallus and normal urethral meatus. Both testes were descended and were symmetric and nontender, with no intratesticular masses. A nontender, firm 0.8 cm mobile mass was palpated superior to the right testicle and was noted to be completely separate from the testis. Scrotal examination was otherwise normal. The patient had no inguinal lymphadenopathy bilaterally, and his abdominal examination was normal.

**Figure 1:**
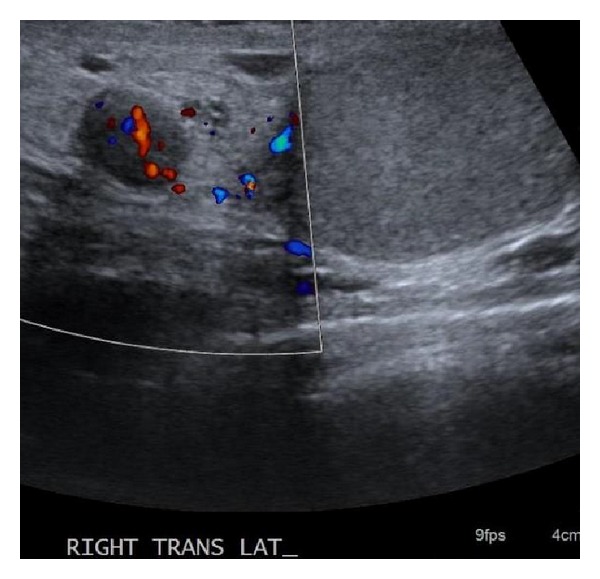
Scrotal sonogram demonstrating a 0.7 × 0.8 × 0.6 cm hypoechoic, vascular, heterogeneous lesion superior to the normal right testicle.

Scrotal sonography confirmed a 0.7 × 0.8 × 0.6 cm hypoechoic, vascular, slightly heterogeneous lesion superior to the right testicle (Figure 1). The testes and epididymides were normal bilaterally. Quantitative serum beta-human chorionic gonadotropin, alpha-fetoprotein, and lactate dehydrogenase were within normal limits.

**Figure 2:**
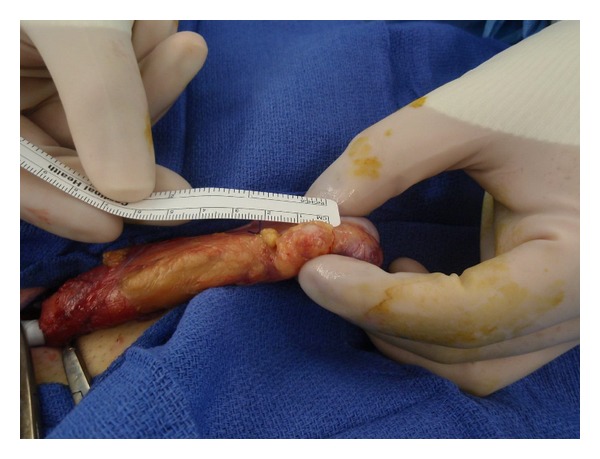
Intraoperative photograph demonstrating complete mobilization of the right spermatic cord, with proximal control achieved with a Penrose drain. The paratesticular lesion is depicted superior to the right epididymis, with a moderate amount of fat surrounding the mass.

Despite a low suspicion for malignancy, surgical exploration was performed through a right inguinal approach with proximal control of the spermatic cord. The lesion was identified superior to the right epididymis and was noted to be mobile and completely separate from the epididymis, vas deferens, and testis (Figure 2). A moderate amount of fat surrounded the lesion. The mass was removed intact along with a rim of fat circumferentially. Frozen section analysis revealed an encapsulated lesion with a predominance of spindle cell proliferation and lymphocytic aggregates, with no malignant features. Given the absence of indication for immediate radical orchiectomy, the spermatic cord was unclamped, and the testis was replaced into the scrotum.

**Figure 3:**
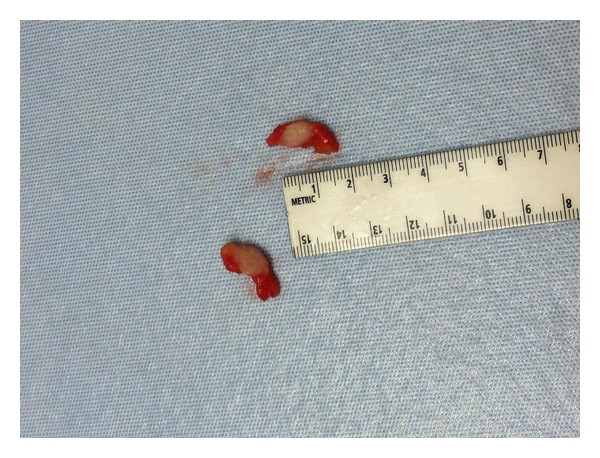
Excised, bivalved lesion revealing a white, smooth nodule measuring 1.3 × 0.6 × 0.3 cm with surrounding fat.

**Figure 4:**
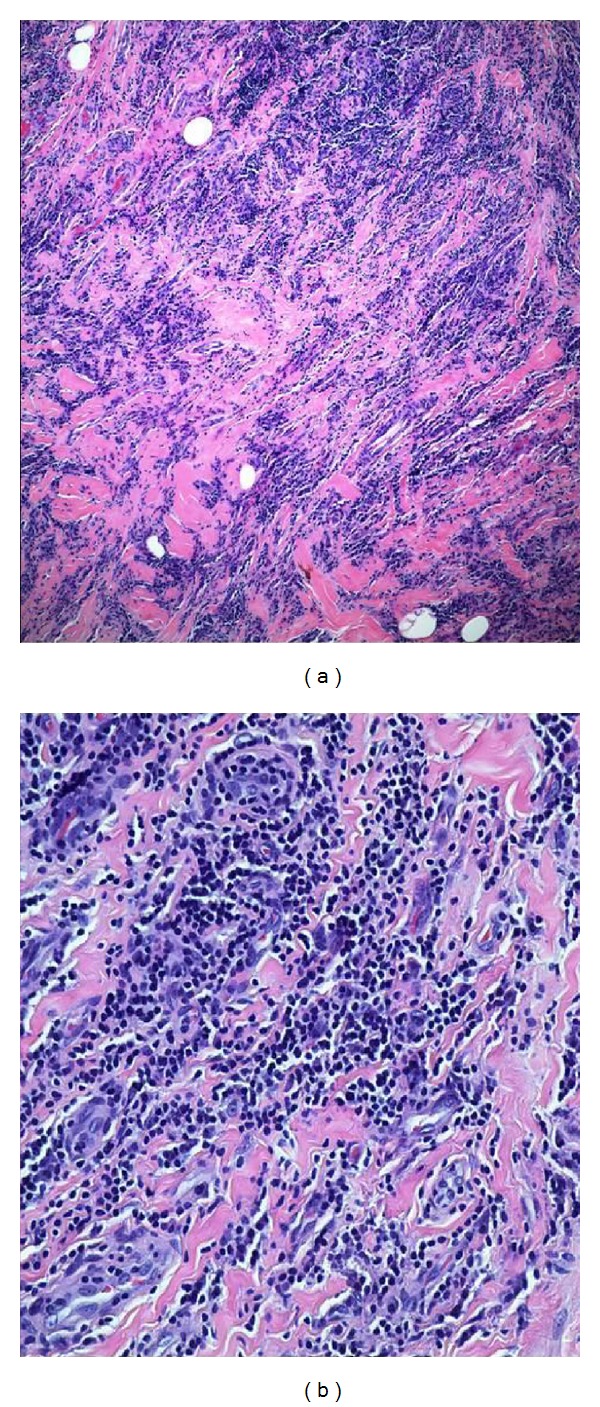
Microscopic low-power (a) and high-power (b) views demonstrating fibrous proliferation (pink staining) and lymphoplasmacytic infiltrate (blue staining), with no cytologic atypia, mitotic activity, or necrosis.

Final gross pathologic analysis revealed a white, smooth nodule measuring 1.3 × 0.6 × 0.3 cm (Figure 3). Microscopically, the mass demonstrated benign fibrous proliferation with lymphoplasmacytic infiltrate, consistent with IMT. No cytologic atypia, mitotic activity, or necrosis was present (Figure 4). The mass was noted to be completely excised. Immunohistochemistry revealed negative staining for myogenin, cytokeratin MNF116, calretinin, mast cell tryptase, and anaplastic lymphoma kinase (ALK). Immunohistochemical stains for IgG and IgG4 showed only scattered IgG4-positive plasma cells. Staining for kappa and lambda light chains confirmed the absence of monoclonality in the plasma cells.

The patient recovered uneventfully. Physical examination three months postoperatively revealed a well-healed incision and no evidence of recurrence.

## 3. Discussion

IMT is a rare spindle tumor that has been assigned many names, including inflammatory pseudotumor, plasma cell pseudotumor, xanthomatous pseudotumor, atypical myofibroblastic tumor, atypical fibromyxoid tumor, pseudosarcoma, plasma cell granuloma, and fibrous pseudotumor [2–6]. This tumor has been described in multiple anatomic sites, including the lung, gastrointestinal tract, retroperitoneum, central nervous system, extremities, and the genitourinary tract. There is a 2 : 1 to 3 : 1 male predominance, and the majority of cases occur in young adults [3]. IMT is typically slow-growing and rarely demonstrates aggressive behavior [3].

IMT is the second most common paratesticular mass after adenomatoid tumor and comprises about 6% of paratesticular lesions [1]. Approximately two-thirds involve the tunica vaginalis, while the remainder may be associated with the tunica albuginea, epididymis, or spermatic cord [2, 7]. Although the peak incidence occurs in the third decade of life, all age groups may be affected [1]. However, only approximately four cases have been reported in patients younger than 18 years of age [2]. Patients typically present with a painless scrotal mass ranging from 0.5 to 8 cm in size, with the largest tumor reported to be 25 cm [2]. A history of scrotal trauma, surgery, or infection can be elicited in approximately one-third of cases [1], and an associated hydrocele is present in nearly 50% of affected patients [2].

Grossly, IMT is typically a circumscribed, firm, gray-white or yellow lesion [3]. Foci of necrosis, hemorrhage, calcification, and cystic changes are rare [8]. Histologically, these lesions are characterized by a predominance of myofibroblast and fibroblast spindle cells in a collagenous or myxoid matrix, with a mixed inflammatory infiltrate mainly comprised of plasma cells, lymphocytes, and occasional eosinophils [3, 6]. Necrosis, high mitotic activity, and increased pleomorphism are typically absent [2]. Immunohistochemical staining is reportedly positive for vimentin (95% to 100%), desmin (5% to 80%), smooth muscle actin (48% to 100%), muscle specific actin (62%), ALK (50%), and keratin (10% to 89%) [3]. These lesions usually show negative immunoreactivity for myoglobin or S100 protein [6].

The etiology of IMT remains unclear. It is postulated that these lesions may represent an exaggerated reparative response to trauma, chronic irritation, or infection [6]. Epstein-Barr virus, mycobacterium avium-intracellulare, and human herpesvirus 8 have been cultured from pulmonary, splenic, and hepatic IMT; however, these infectious agents have not been identified in paratesticular IMT [3]. Autoimmune etiology has also been suggested in tumors outside of the genitourinary tract [3].

A neoplastic etiology is suggested in some cases that demonstrate genetic aberrations in the short arm of chromosome 2 in region p21–p23, involving a 2p23 rearrangement in the* ALK* gene. The human* ALK* gene encodes ALK, a tyrosine kinase receptor and member of the insulin growth factor receptor superfamily [3].* ALK* gene alterations lead to fusion with other genes, and several translocations have been reported [3].

Some pseudotumors demonstrate elevated levels of intralesional IgG4-expressing plasma cells. Thus, it has also been proposed that IMT may represent a localized form of IgG4-related sclerosing disease, which refers to a group of disorders characterized by chronic fibroinflammatory processes at various anatomic sites [9].

Scrotal ultrasonography is usually the initial diagnostic modality and assists in distinguishing between an intratesticular versus extratesticular lesion as well as a solid versus cystic mass. IMT may appear as a hypoechoic or hyperechoic lesion, depending on its degree of collagen, calcifications, or fibroblasts [7]. Unlike intratesticular masses, the majority of paratesticular lesions are benign. The differential diagnosis of a paratesticular mass is extensive and includes benign lesions such as adenomatoid tumor, IMT, cystadenoma, hydrocele, spermatocele, varicocele, hernia, tunica albuginea cyst, adrenal rest, splenogonadal fusion, and tubular ectasia of the rete testis [10, 11], as well as rare malignant lesions including inflammatory fibrosarcoma, leiomyosarcoma, liposarcoma, rhabdomyosarcoma, and cystadenocarcinoma [4, 6].

Radiologic studies usually cannot differentiate between benign and malignant lesions; therefore, wide surgical excision is the treatment of choice for paratesticular IMT, with testis sparing when possible. Frozen section assessment is useful for intraoperative decision making. A recent study demonstrated that frozen section assessment aided in preventing unnecessary radical orchiectomy in 36 out of 43 (83.7%) benign testicular and paratesticular lesions [12]. However, even when benign pathology is suspected, testis sparing may not be possible if the mass is very large or cannot be reliably differentiated from the testis [6]. Therefore, preoperative counseling should address the possibility of radical orchiectomy.

Prognosis after complete surgical resection of paratesticular IMT is excellent. Recurrence rates of 23% to 37% have been reported in abdominal and retroperitoneal IMT [5]; however, to our knowledge, no recurrence has been reported after complete excision of paratesticular lesions.

## 4. Conclusion

Although rare, IMT should be considered in the differential diagnosis of paratesticular lesions. The treatment of choice is wide excision with testis sparing when possible; however, preoperative counseling should address the possibility of radical orchiectomy. Frozen section assessment is useful for intraoperative decision making. Prognosis after complete excision of paratesticular inflammatory pseudotumor is excellent. To our knowledge, no recurrence has been reported; however, continued follow-up is recommended.

## Abbreviations

IMT: Inflammatory myofibroblastic tumor
ALK: Anaplastic lymphoma kinase.
